# Circular RNA expression profiles and functional predication after restraint stress in the amygdala of rats

**DOI:** 10.3389/fnmol.2024.1381098

**Published:** 2024-04-15

**Authors:** Chuan Wang, Qian Wang, Guangming Xu, Zhaoling Sun, Dong Zhang, Chunling Ma, Yingmin Li, Di Wen, Xiaojing Zhang, Bin Cong

**Affiliations:** ^1^College of Forensic Medicine, Hebei Key Laboratory of Forensic Medicine, Collaborative Innovation Center of Forensic Medical Molecular Identification, Hebei Medical University, Shijiazhuang, China; ^2^Department of Forensic Medicine, The National Police University for Criminal Justice, Baoding, China; ^3^College of Integrative Medicine, Hebei University of Chinese Medicine, Shijiazhuang, China; ^4^Hainan Tropical Forensic Medicine Academician Workstation, Haikou, China

**Keywords:** circular RNA, restraint stress, amygdala, biomarker, RNA-seq

## Abstract

Prolonged or repeated exposure to stress elevates the risk of various psychological diseases, many of which are characterized by central nervous system dysfunction. Recent studies have demonstrated that circular RNAs (circRNAs) are highly abundant in the mammalian brain. Although their precise expression and function remain unknown, they have been hypothesized to regulate transcriptional and post-transcriptional gene expression. In this investigation, we comprehensively analyzed whether restraint stress for 2 days altered the circRNA expression profile in the amygdala of male rats. The impact of restraint stress on behavior was evaluated using an elevated plus maze and open field test. Serum corticosterone levels were measured using an enzyme-linked immunosorbent assay. A total of 10,670 circRNAs were identified using RNA sequencing. Ten circRNAs were validated by reverse transcription and quantitative polymerase chain reaction analysis. Gene ontology and Kyoto encyclopedia of genes and genomes pathway analyzes supported the notion that genes associated with differentially expressed circRNAs are primarily implicated in neuronal activity and neurotransmitter transport. Moreover, the three differentially expressed circRNAs showed high specificity in the amygdala. Overall, these findings indicate that differentially expressed circRNAs are highly enriched in the amygdala and offer a potential direction for further research on restraint stress.

## 1 Introduction

Stress, an increasingly pervasive condition in contemporary culture, is a non-specific response to diverse nocuous agents. Initially, Selye described stress as a general adaptation syndrome (Selye, 1936). Through exhaustive studies, scholars have concluded that moderate stress is fundamentally a defensive adaptive response of organisms for survival (Janak and Tye, 2015). However, excessive or persistent stress can induce homeostasis disorders and neuronal remodeling. For example, in the event of major disasters like earthquakes, mining accidents, and significant traffic incidents, individuals might endure extended periods of being confined in limited spaces or immobilized. It may potentially contribute to the development of several mental illnesses, including depression, anxiety, and post-traumatic stress disorder (PTSD) (Ganzel et al., 2008; Roozendaal et al., 2009; Cénat et al., 2020; Ma et al., 2021). The central nervous system is critical for modulating stress responses (Russell and Lightman, 2019).

The amygdala, a principal limbic system component, regulates physiological and behavioral responses to stress (Maren and Holmes, 2016; Krabbe et al., 2018). It is essential in emotion regulation, fear memory acquisition, and extinction (Zhang et al., 2021). Numerous studies have established that restraint stress can trigger structural remodeling of the amygdala and alterations in the organism’s physiological states and behaviors (Masneuf et al., 2014; Chattarji et al., 2015). Prior research has demonstrated that restraint stress in rats is strongly associated with neuronal and blood–brain barrier damage in the amygdala and the emergence of depression and anxiety (Wang et al., 2019; Xu et al., 2019). However, these phenomena occurring in the amygdala after stress response remain unexplored.

Circular RNAs (circRNAs), a novel member of noncoding RNAs (ncRNAs), are a distinct class of regulatory RNA, possessing a covalently closed loop structure without the 5′ and 3′ termini (Salzman et al., 2012; Kristensen et al., 2019). These are highly abundant in eukaryotes, and many are evolutionarily conserved (Memczak et al., 2013; Salzman et al., 2013). CircRNAs are thus considerably insusceptible to degradation by exonucleases and are more stable than their linear RNA molecules (Suzuki and Tsukahara, 2014; Li et al., 2018). Recent studies have reported that circRNAs exhibit tissue- and temporal stage-specific expressions in drosophila, mice, rats, and humans (Westholm et al., 2014; Xia et al., 2016; Sharma D. et al., 2021). Notably, certain circRNAs are significantly enriched in neuronal tissues (Chen and Schuman, 2016). These attributes of circRNAs imply that they have unique biological functions rather than being a by-product of pre-mRNA processing. Over the last decade, many researchers have revealed that circRNAs can function as microRNA sponges, interact with RNA-binding proteins (RBPs), modulate alternative splicing and transcription, and even translate into polypeptides (Patop et al., 2019; Huang et al., 2020; Xiao et al., 2022). Accumulating evidence indicates that circRNAs are involved in neurological disorders, including ischemic brain injury, Alzheimer’s disease, and PTSD (Mo et al., 2020; Su et al., 2021; Du et al., 2022). Due to their specific features, circRNAs can serve as diagnostic and prognostic biomarkers for some central nervous system disorders. However, few studies have suggested the potential impact of circRNAs on the pathophysiological mechanisms in the amygdala under various conditions of restraint stress.

In this study, we exposed male rats to sustained restraint stress for 2 days, which simulated the prolonged spatial confinement and immobility experienced by humans, and analyzed whether sustained restraint stress altered the circRNA expression profile in the amygdala of rats. This study aimed to enhance our comprehension of restraint stress-associated circRNA profiles in the amygdala, offering new indications into circRNAs as potential targets in the pathophysiological processes of restraint stress.

## 2 Materials and methods

### 2.1 Animals

The entire animal experimental protocol followed the National Institutes of Health Guide for the Care and Use of Laboratory Animals. It was also established by the Local Committee of Animal Care, Use, and Protection of Hebei Medical University. Male Sprague–Dawley rats (weighing 200–250 g and aged 6–8 weeks) were obtained from Beijing Vital River Laboratory Animal Technology Co., Ltd. (Beijing, China). Rats were group-housed in an environment with controlled temperature (22 ± 1°C) and humidity (55% ± 5%) and provided *ad libitum* access to water and forage under a standard 12-h light/dark cycle. The rats were fed adaptively for 7 days before any experimental procedures.

### 2.2 Stress protocol

Adult male Sprague–Dawley rats were randomly divided into three groups (*n* = 9 for each group). Rats were confined within an acrylic tube (20 cm length, 5.5 cm inner diameter) to immobilize their bodies constantly for 2 days (restraint stress group, RS). Rats were fasting for 2 days without restraint stress (control group). Rats had free access to autoclaved food and water for 2 days (naive group).

### 2.3 Body weight monitoring

The body weights of all rats were measured throughout the experiment.

### 2.4 Behavioral assessment

The animals were acclimatized to the experimental room for 1 h before the experimental procedures.

#### 2.4.1 Open field test (OFT)

The OFT was performed to assess the locomotor function of the animals. During the experiment, animals were separated from each other, and the test room was kept darkly lit and noise isolated. The apparatus comprised a black acrylic box (100 × 100 × 40 cm) and a video tracking system. Each animal was initially placed in the central arena and allowed to explore the space for 5 min. Spontaneous activity was recorded and analyzed using Noldus EthoVision XT software (Version 16.0, Noldus Information Technologies, Wageningen, The Netherlands). The travel distance, time spent in the center area, and total travel distance were calculated (Shi et al., 2023). The apparatus was cleaned with 70% ethyl alcohol to remove excrement and odor after each trial.

#### 2.4.2 Elevated plus maze (EPM)

An elevated plus maze test was used to measure anxiety levels (Gholami Mahmoudian et al., 2023). The maze consisted of two open arms without walls (50 × 10 × 0.5 cm), two enclosed arms with walls (50 × 10 × 40 cm), and a central area (10 × 10 cm) elevated at 50 cm height. The same arms were opposite to each other. Rats were moved in the central area toward one of the open arms and allowed to explore the maze freely for 5 min. After each experiment, the apparatus was thoroughly cleaned with 70% ethanol to eliminate excrement and odors. Travel distance, time spent in the open arms, and total travel distance were calculated (Shanghai Jiliang Software Science & Technology Co., Ltd., Shanghai, China) (Kraeuter et al., 2019; Fang et al., 2021). The lower the time spent and distance moved in the open arms, the higher the anxiety state. After each trial, the maze was cleaned with 70% ethanol to remove excrement and odor.

### 2.5 Tissue collection and RNA isolation

After the procedure, all rats were deeply anesthetized with an intraperitoneal injection of 10% chloral hydrate (4 mL/kg). The rats were sacrificed, and their brains were removed and immediately placed on ice. According to the rat stereotaxic map by George Paxinos (2007), bilateral amygdala tissue was isolated. The amygdala tissue was preserved in RNAlater overnight at 4°C, then transferred to −80°C until processing. Blood samples were collected via cardiac puncture and centrifuged at 1000 × g and 4°C for 15 min. Serum was extracted in 1.5-mL EP tubes and stored at −80°C for further CORT quantification. Sections of heart, kidney, liver, lung, skeletal muscle, spleen, and testis tissues from the same location of each rat were cut and preserved in RNAlater overnight at 4°C and then transferred to −80°C until processing.

Pools of three amygdala were isolated from three rats from the same group to fulfill the sequencing criterion. Total RNA was extracted from frozen tissues using TRIzol reagent (Thermo Scientific, United States) following the manufacturer’s standard protocols. RNA quantification of each sample was performed by NanoDrop 1,000 (NanoDrop Technologies). RNA integrity was measured using the RNA Nano 6,000 Assay Kit on the Bioanalyzer 2,100 system (Agilent Technologies, CA, United States). The isolated RNAs were cryopreserved at −80°C until use.

### 2.6 Enzyme-linked immunosorbent assay

Serum corticosterone (CORT) levels were detected using an enzyme-linked immunosorbent assay (ELISA) kit (Arigo, ARG80652, Hsinchu, Taiwan, China), following the manufacturer’s instructions.

### 2.7 Library preparation and sequencing

RNA (5 μg) from amygdala tissue was used as the input material for RNA sample preparation. Ribosomal RNA was extracted from total RNA with Epicentre Ribozero^™^ rRNA Removal Kit (Epicentre, United States). The rRNA-free residue was eliminated by ethanol precipitation. Subsequently, linear RNA was fully digested with 3 U of RNase R (Epicentre, United States) per μg of RNA. Sequencing libraries were produced according to NEBNext® Ultra™ Directional RNA Library instructions. Finally, libraries were sequenced using the Illumina HiSeq 4,000 platform to generate 150-bp paired-end reads at Novogene Co., Ltd. (Beijing, China). Raw FASTQ files from the RNA-seq data were deposited at the NCBI Sequence Read Archive (BioProject: PRJNA1071307).

### 2.8 Identification of circRNAs

Sequencing results were processed further, and circRNA candidates were identified using find_circ (Memczak et al., 2013) and CIRI2 software (Gao et al., 2015). Normalization of contig counts was accomplished by evaluating transcripts per million (TPMs). Normalized expression level = (read counts × 1,000,000)/lib size (lib size is the sum of circRNA read counts).

To ascertain the indispensable circRNAs responsible for restraint stress in the rat amygdala, a differential expression analysis was conducted using the DESeq2 R package (Love et al., 2014). Benjamini and Hochberg’s procedures were used to adjust the *p* values for controlling the false discovery rate. Genes with adjusted *p*-values (*p*adj) < 0.05 may be discovered by DESeq2. Transcripts with large differences between the control and restraint stress groups were considered differentially expressed. The volcano plot based on adjusted *p*-values was created using GraphPad software. Principal component analysis (PCA) depends on the expression levels of genes required for statistical computing and graphics (Metsalu and Vilo, 2015).

### 2.9 Quantitative real-time PCR validation

Ten circRNAs were selected to validate circRNA expressions. Total RNA was extracted from the tissues of adult rats in each sample and reverse transcribed to synthesize complementary DNA (cDNA) using the PrimeScript RT reagent Kit with gDNA Eraser (Takara Bio, Shiga, Japan) following the manufacturer’s instructions. Reaction amplification and quantification were performed with SYBR® Premix Ex Taq II (Takara Bio, Shiga, Japan) on a 7,500 System (Applied Biosystems). Subsequently, the PCR amplification products were assayed by gel electrophoresis, and the sequence was detected by bidirectional Sanger sequencing (Sangon Biotech, Shanghai, China). Considering the unique structure of circRNAs, divergent primers were designed for each candidate across the estimated back spliced junction. Primers were designed with Primer Premier 6, evaluated by Oligo 7, and synthesized by Sangon Biotech (Shanghai, China). Primer specificity was verified using NCBI primer BLAST. For accuracy of the results, glyceraldehyde 3-phosphate dehydrogenase (GAPDH), beta-actin (ACTB), acidic ribosomal phosphoprotein P0 (ARBP), and succinate dehydrogenase complex, subunit A (SDHA) were used as internal controls to quantify the relative expression levels (Langnaese et al., 2008; Gubern et al., 2009; Zhang et al., 2014). These reactions were conducted with three technical replicates. Relative transcript levels were quantified using the 2^−ΔΔCT^ approach (Rao et al., 2013). An extended ΔCT-method was utilized to enhance the reproducibility of relative quantification in RT-qPCR analysis (Bartosch et al., 2014). The primer amplification efficiency of 10 candidate circRNAs and 4 reference genes ranged from 90.599 to 109.132%, with an R^2^ range of 0.980–0.998 (Supplementary Table S1). The details of primers used in this study are presented in Supplementary Table S2.

### 2.10 Function prediction of validated differentially expressed circRNAs

As a crucial initiative in bioinformatics, Gene ontology (GO) and Kyoto encyclopedia of genes and genomes (KEGG) pathway enrichment analyzes were performed to annotate host genes derived from differentially expressed circRNAs between the two conditions. GO enrichment analysis was conducted with the GOseq R package, and the length bias of the gene was rectified (Young et al., 2010). KEGG pathway enrichment analysis was executed using KOBAS-i. The analysis investigated the statistical enrichment of the differentially expressed circRNA host genes (Bu et al., 2021). A *p*-value < 0.05 was considered statistically significant for differentially expressed genes in GO terms and KEGG pathways.

### 2.11 circRNA-miRNA interaction research

circRNAs possess multiple miRNA binding sites that can interfere with functional miRNAs by acting as miRNA sponges (Hansen et al., 2013). For a deeper understanding of the process of miRNA interaction with circRNA, we created circRNA-miRNA interaction networks using miRanda software from the total predicted. We excluded those with match scores above 140 and minimum free energy below −10 kcal/mol to enhance the reliability of our findings. The interaction network of the 10 circRNAs was shown using Cytoscape 3.10.0.

### 2.12 Construction of differentially expressed circRNAs in different tissues

To determine the expression characteristics of circRNAs in different rat tissues, we evaluated a public total RNA-seq dataset in the Ensemble Database (Yu et al., 2014) and detected six differentially expressed circRNAs by qRT-PCR. In addition, we examined eight rat tissues, including the amygdala, heart, kidney, liver, lung, skeletal muscle, spleen, and testis tissue samples. qRT-PCR was conducted as described previously.

### 2.13 Statistical analysis

Data was analyzed using GraphPad Prism 9.0.0 (GraphPad Software, Inc., La Jolla, CA, United States) and expressed as the mean ± standard deviation (SD). The correlation between circRNA expression levels and behavioral indicators was assessed using Pearson’s correlation coefficient (Pearson’s r > 0.5 and *p* < 0.05). Data normality was examined by the Shapiro–Wilk test. Comparisons of two independent groups were performed utilizing Student’s *t*-test. Normally distributed data were analyzed using one-way ANOVA followed by Tukey’s post-hoc test for multiple comparisons. Non-normally distributed data from different groups were compared using the Kruskal-Wallis test and Dunn’s multiple comparison post-test. *p* < 0.05 was considered statistically significant.

## 3 Results

### 3.1 Overview of body weight and CORT levels

**Figure 1:**
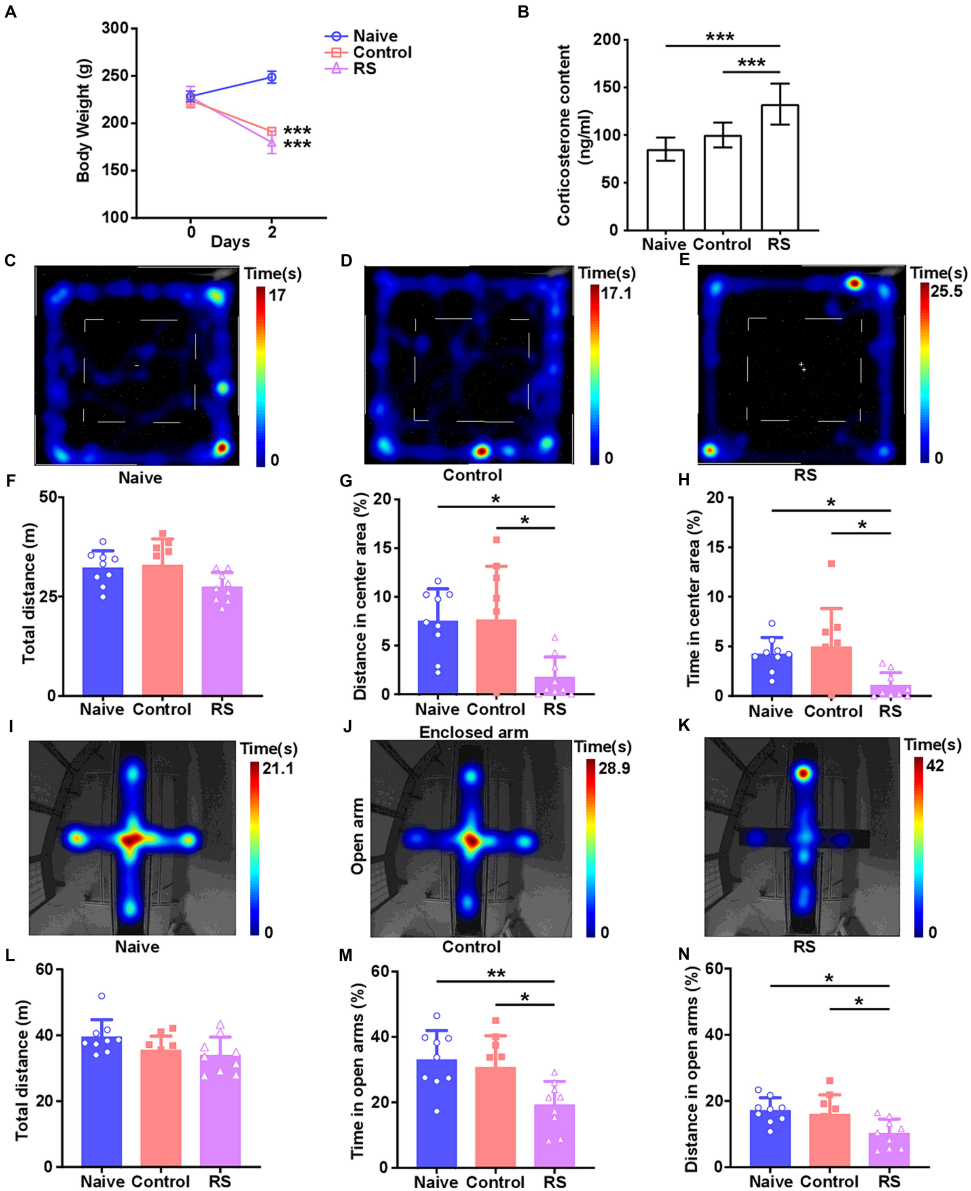
Effect of restraint stress on body weight, serum CORT levels, and behavioral changes in rats. **(A)** Compared with the naive group, the restraint stress and control groups remarkably decreased body weight gain. **(B)** Serum levels of CORT measured after naive, control, and restraint stress procedures in rats. **(C–E)** Heatmap tracks of naive, control, and restraint stress rats in the OFT. **(F–H)** Restraint stress reduced the time spent and traveled distance in the center area but had no impact on the total traveled distance in naive. **(I–K)** Characteristic movement tracks of naive, control, and restraint stress rats in the elevated plus maze. **(L–N)** Restraint stress reduced the time spent and traveled distance in open arms but had no impact on the total traveled distance in naive. Results are presented as mean ± SD. Data were analyzed using one-way ANOVA followed by Tukey’s post-hoc test. *p < 0.05, **p < 0.01, and ***p < 0.001 contrasted with the naive.

To accurately investigate the effect of the restraint stress model on rats, we measured body weight and serum CORT levels. Compared with the naive group, rats in the restraint stress and control groups exhibited a significant decrease in body weight [*F* (2,24) = 192.2, *p* < 0.001; Figure 1A]. There was no clear difference in body weight among the three groups upon the commencement of the trial. Meanwhile, a significant increase in serum CORT levels was observed in restraint-stressed rats compared with naives [*F* (2,24) = 20.46, *p* < 0.001]. There was no obvious difference between the control and naive groups (Figure 1B). These results suggest that the restraint stress model in rats was successfully established.

### 3.2 Effect of restraint stress on rat behaviors

The OFT and EPM were conducted to evaluate anxiety-like behaviors and the ability to spontaneously explore activities in restraint-stressed rats. In the OFT, a significant decrease in the percentage of distance traveled [*F* (2,24) = 6.656, *p* = 0.005] and time spent [*F* (2,24) = 5.837, *p* = 0.009] in the center area was observed in restraint-stressed rats at 2 days, compared with the naive and control groups. There was no clear difference in the total distance traveled between the three groups (Figures 1C–H). Based on the OFT results, a significant reduction in the proportion of time spent [*F* (2,24) = 6.472, *p* = 0.006] and distance traveled [*F* (2,24) = 5.448, *p* = 0.01] in the open arms in the EPM was observed for restraint-stressed rats at 2 days, compared with the naive and control rats. There was no significant difference in the total travel distance between the three groups (Figures 1I–N). During the observation period, fasting did not affect rats’ exploratory behavior or locomotor activity compared with the naive group. These findings indicate that restraint stress induces anxiety-like behavior.

### 3.3 Description quality of circular RNA-seq

CircRNA profiles from the amygdalar tissues of restraint stress for 2 days and control rats were obtained by RNA-seq. We manufactured and sequenced six ribosomal RNA (rRNA)-depleted libraries, a restraint stress two-day group (c1, c2, c3), and a control group (d1, d2, d3). After eliminating adapter-containing, poly-N-containing, and low-quality reads, the number of clean reads obtained from each library was 52,360,826–69,249,082. The percentage of clean reads/raw reads in the different libraries was greater than 97.82%. The GC content is a critical component in species assessment. The distribution of the GC content of all samples was 58.33–60.33%, without significant difference. The sequence information and quality assessment of each library are presented in Supplementary Table S3.

**Figure 2:**
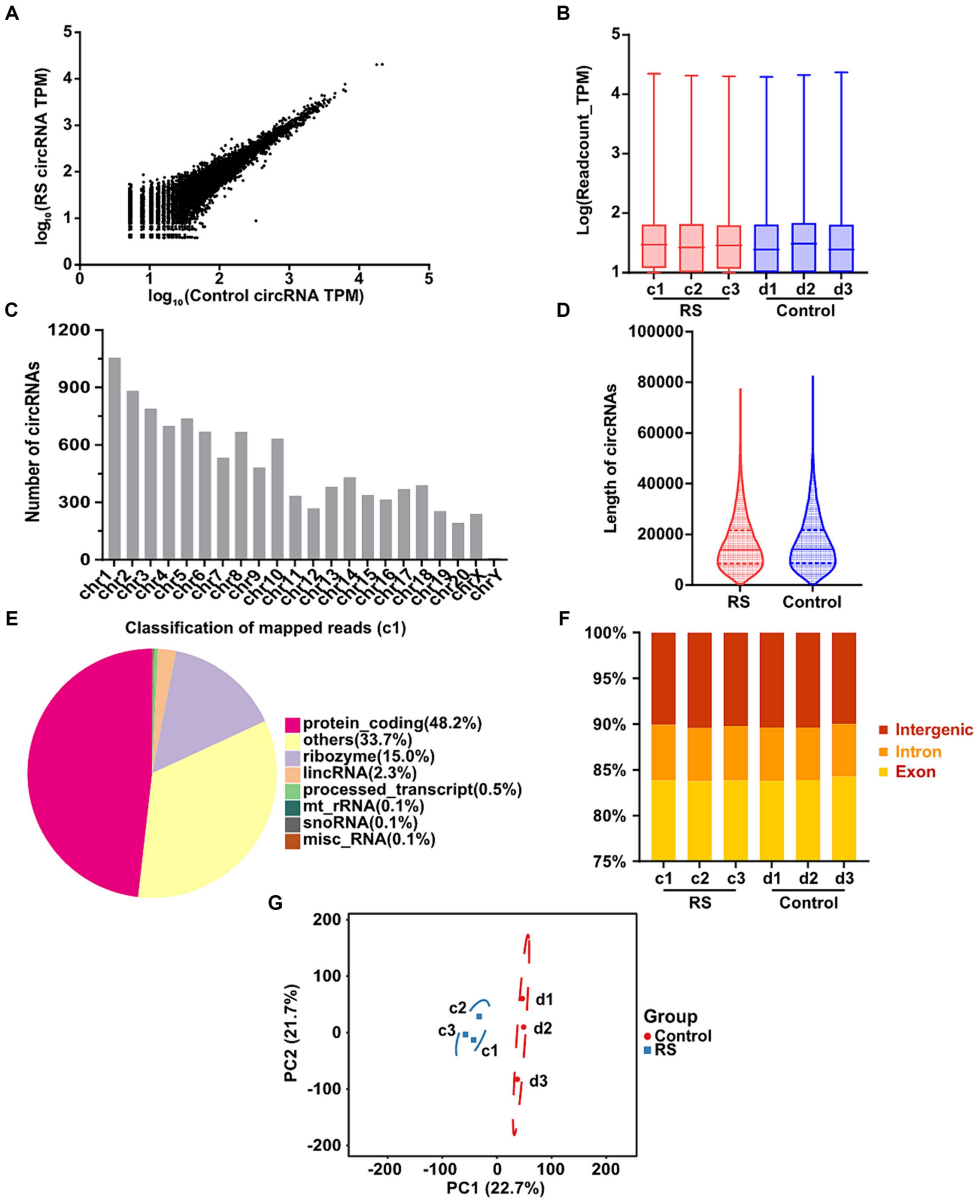
Landscape of circRNAs identified in the amygdala of rats in the control and restraint stress samples. **(A)** Expression of circRNAs is shown by a scatter plot between the control and restraint stress groups. **(B)** Density distribution of the TPM values of circRNAs in all libraries. **(C)** Chromosome locations of all unique circRNAs identified by sequencing. **(D)** Length distribution of circRNAs in the control and restraint stress samples. **(E)** Classification of mapped reads using Sample a1. **(F)** Fractions of exons, introns, and intergenic circRNA species in each sample. **(G)** Principal component analysis of circRNAs identified in the control and restraint stress samples.

In total, 10,670 distinct circRNAs were detected between restraint stress two-day and the control groups (Figure 2A). The TPM value distribution of the total circRNAs exhibited that the expression patterns of circRNAs in all samples were highly consistent (Figure 2B). CircRNAs were derived from different chromosomes, distributed on chromosomes 1–20 and sex chromosomes. Chromosome 1 had the greatest number of circRNA species, whereas chromosome Y had the lowest number of circRNA species (Figure 2C). Most identified circRNAs were less than 21,000 nucleotides (nt) in length (Figure 2D). CircRNAs were distributed in various genomic regions but mainly in protein-coding regions. Approximately 50% of circRNAs originated from protein-coding regions (Figure 2E). We also found that exons and introns constituted the majority (approximately 90% in each sample) (Figure 2F). Consequently, no obvious differences were observed in the constitution of the circRNAs in any of the samples. Principal component analysis (PCA) based on circRNA expression levels showed significant differences between the restraint stress two-day and control groups (Figure 2G).

### 3.4 Differential expression analysis and validation of circRNAs in the amygdala

**Figure 3:**
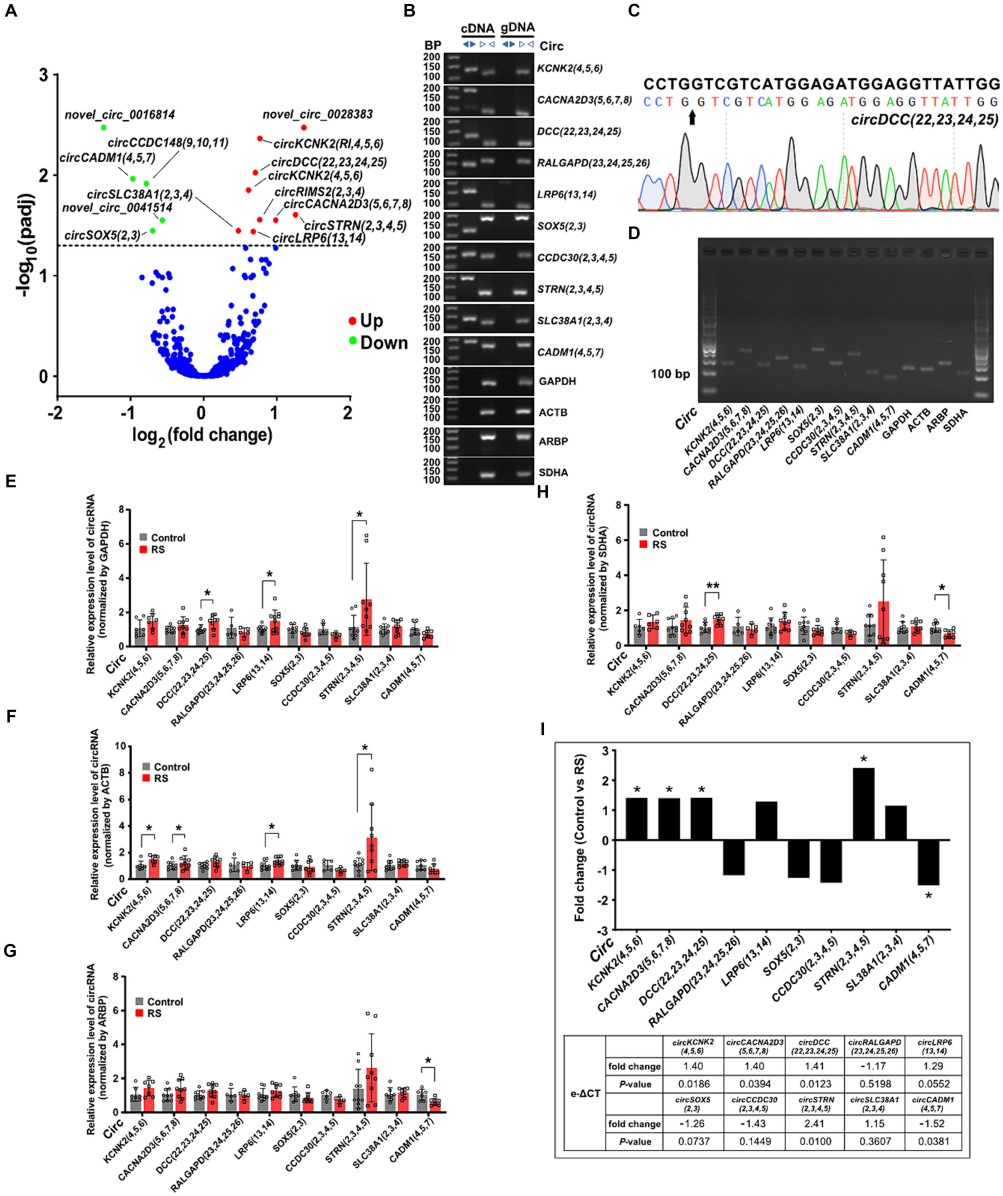
Differential expression and validation of circRNAs in the amygdala. **(A)** Volcano plots of differentially expressed circRNAs detected in the restraint stress two-day group. The log of fold-change (base 2) is plotted on the X-axis, and the negative log of the adjusted *p*-values (base 10) is plotted on the Y-axis. Each dot on the graph represents one circRNA; blue dots represent no significant differences in circRNAs; and red and green dots represent upregulated and downregulated circRNAs, respectively (*p*adj < 0.05). **(B)** The presence of circRNAs in the amygdala was validated using qRT-PCR and agarose gel electrophoresis. Divergent primers only amplified circRNAs from cDNA, and no results were obtained from gDNA or linear control genes. bp: size markers. **(C)** Sanger sequencing of *circDCC(22,23,24,25)* revealed a back-splice junction sequence. The black arrow indicates the backsplice junction. **(D)** Electropherogram showing qRT-PCR amplification product bands of target circRNAs and control genes in the amygdala. **(E–H)** Relative circRNA levels were determined by qRT-PCR in the restraint-stressed and control groups normalized by four control genes (GAPDH, ACTB, ARBP, and SDHA), respectively. RS: restraint stress, *p < 0.05, **p < 0.01 compared with the control. **(I)** The changes in target gene expression levels (expressed as fold change) were calculated using the e-ΔCT method. A comparison between the fold changes in circRNA expression levels in the restraint-stressed and control groups was made using e-ΔCT method with GAPDH, ACTB, ARBP, and SDHA. *: *p*-values <0.05.

We performed expression profiling to verify whether circRNAs are differentially expressed in the amygdala during restraint stress. The results revealed that 14 circRNAs were differentially expressed two days after restraint stress (compared with the control group, *p*adj < 0.05) (Supplementary Table S4). Nine circRNAs exhibited significant upregulation, whereas five showed considerable downregulation (Figure 3A). CircRNAs were named according to the recommendations (Chen et al., 2023). Ten circRNAs [*circKCNK2(4,5,6)*, *circCACNA2D3(5,6,7,8)*, *circDCC(22,23,24,25)*, *circRALGAPD(23,24,25,26)*, *circLRP6(13,14)*, *circSOX5(2,3)*, *circCCDC30(2,3,4,5)*, *circSTRN(2,3,4,5)*, *circSLC38A1(2,3,4)* and *circCADM1(4,5,7)*] were randomly selected to determine their presence and expression in the amygdala of rats (eight differentially expressed circRNAs and two non-differentially expressed circRNAs). Ten circRNAs were successfully amplified using divergent cDNA primers, whereas genomic DNA (gDNA) and linear control genes (GAPDH, ACTB, ARPB, and SDHA) were not amplified (Figure 3B). The head-to-tail splicing of circRNAs detected in the amygdala was verified using Sanger sequencing (Figure 3C). qRT-PCR amplification analysis of the selected circRNAs and control genes by agarose gel electrophoresis demonstrated that only one band of the expected size was amplified (Figure 3D).

Nine of the ten circRNAs detected by qRT-PCR (normalized to the expression of GAPDH, ACTB, ARBP, and SDHA) were consistent with those measured by RNA-seq, whereas *circSLC38A1(2,3,4)* showed nonconformity (Supplementary Figure S1). These findings indicate that the circRNA expression profiles are highly reproducible and reliable. *CircDCC(22,23,24,25)*, *circLRP6(13,14)*, and *circSTRN(2,3,4,5)* (normalized to the expression of GAPDH) were significantly upregulated in the restraint stress group compared with the control group (Figure 3E). *CircKCNK2(4,5,6)*, *circCACNA2D3(5,6,7,8)*, *circLRP6(13,14)*, and *circSTRN(2,3,4,5)* (normalized to ACTB expression) were significantly upregulated (Figure 3F). *CircCADM1(4,5,7)* (normalized to ARBP expression) was significantly downregulated (Figure 3G). *CircDCC(22,23,24,25)* (normalized to the expression of SDHA) was significantly upregulated, whereas *circCADM1(4,5,7)* was significantly downregulated (Figure 3H). *CircKCNK2(4,5,6)*, *circCACNA2D3(5,6,7,8)*, *circDCC(22,23,24,25), circSTRN(2,3,4,5)* and *circCADM1(4,5,7)* were significantly changed in the restraint stress group compared with the control group calculated by e-ΔCT using: GAPDH, ACTB, ARBP, and SDHA (*p*-values <0.05) (Figure 3I).

Pearson correlation analysis showed that expression of *circKCNK2(4,5,6)* was positively correlated with the time in open arms (r = 0.69, *p* < 0.01), the distance in center area (r = 0.68, *p* < 0.01), and the time in center area (r = 0.61, *p* < 0.01). Expression of *circDCC(22,23,24,25)* was positively correlated with the time in open arms (r = 0.56, *p* < 0.05), the distance in center area (r = 0.68, *p* < 0.01), and the time in center area (r = 0.75, *p* < 0.001). Expression of *circLRP6(13,14)* was positively correlated with the time in open arms (r = 0.57, *p* < 0.05), and the time in center area (r = 0.53, *p* < 0.05) (Supplementary Figure S2).

### 3.5 Go and KEGG pathway analyzes in differentially expressed circRNAs

**Figure 4:**
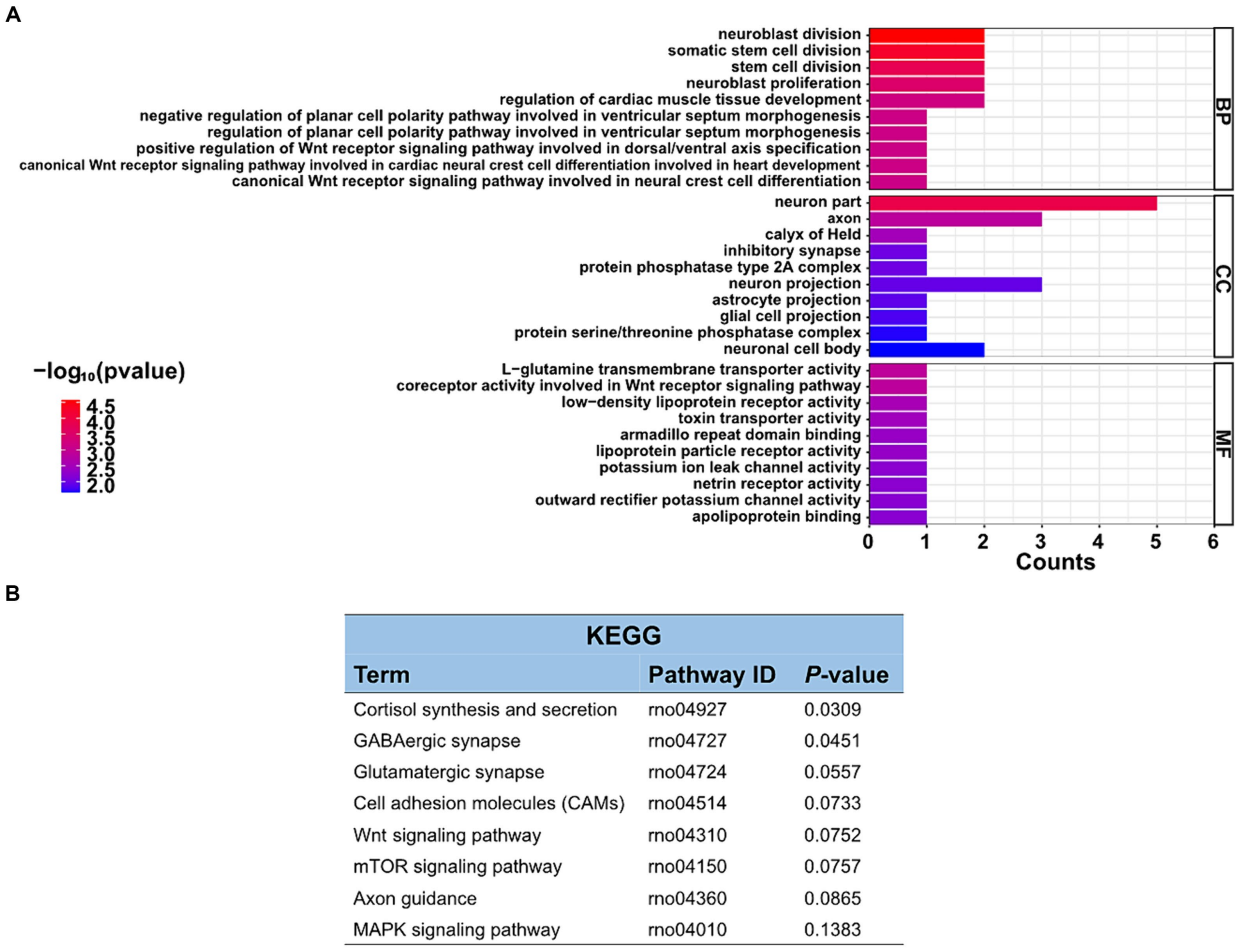
GO enrichment and KEGG pathway analyzes of differentially expressed circRNAs. **(A)** List of the top 10 GO enrichments from biological processes (BP), cellular components (CC), and molecular functions (MF). **(B)** KEGG pathway. Only neuronal-related terms are reported.

The function of these specific circRNAs in regulating restraint stress in the rat amygdala remains unknown. To explore the potential functions of these differentially expressed circRNAs after restraint stress, we performed GO and KEGG pathway analyzes based on the circRNAs’ parental genes. The results showed that 388 GO terms were enriched, including 332 GO BP (biological processes), 21 GO CC (cellular components), and 35 GO MF (molecular functions). The top 10 biological processes, cellular components, and molecular functions terms are illustrated in Figure 4A. Further screening of the terms revealed that certain GO terms were primarily associated with neuronal processes and networks, including neuroblast division, neuron part, axon, neuroblast proliferation, and neuron projection. In the KEGG enrichment analysis, neuron-related regulatory pathways, including cortisol synthesis and secretion, GABAergic synapse, glutamatergic synapse, and axon guidance were successfully screened (Figure 4B). Consequently, the above enrichment analysis indicates that circRNAs may play a crucial role in the pathophysiological process of restraint stress.

### 3.6 Construction of circRNA-miRNA interaction networks

**Figure 5:**
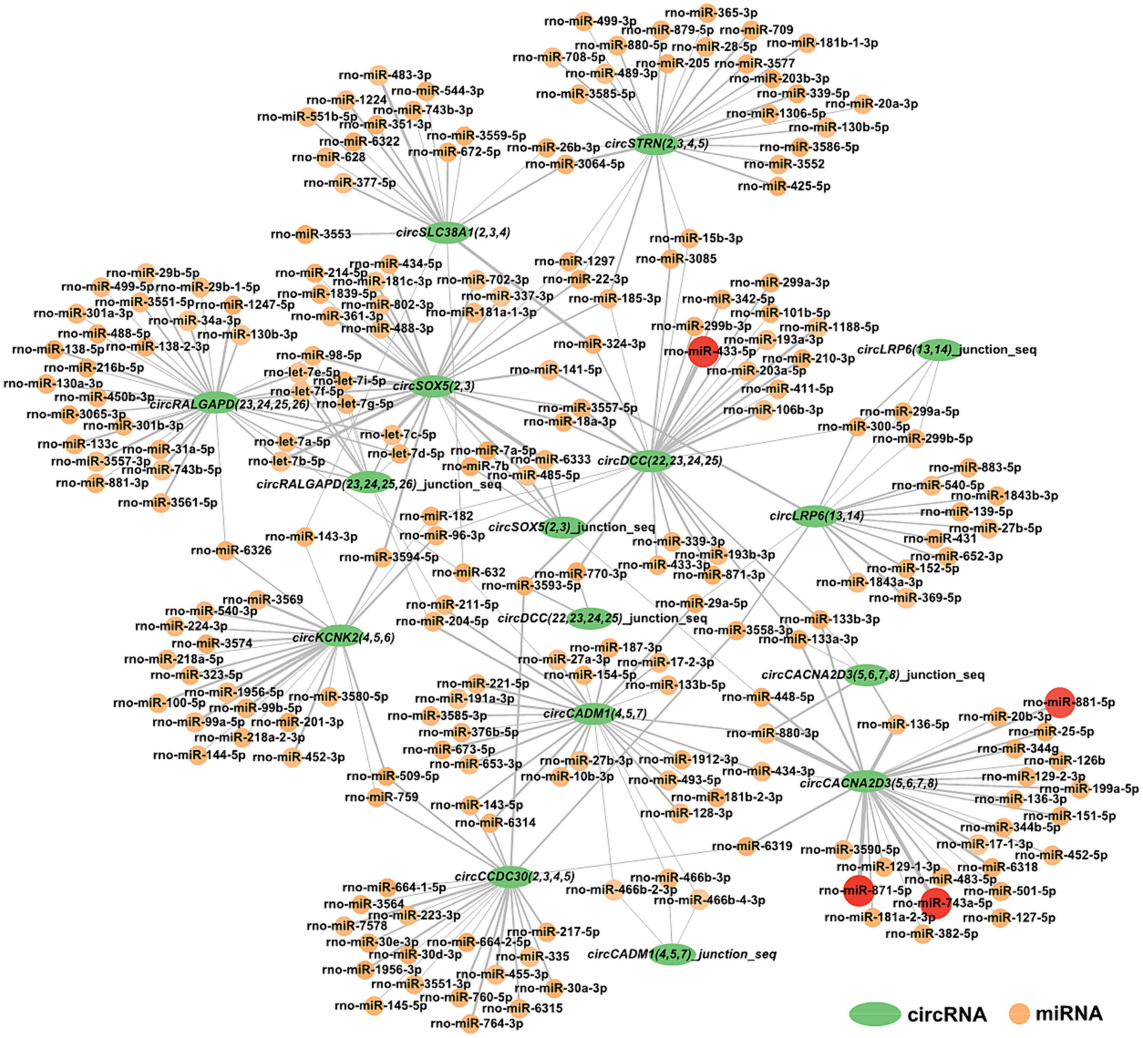
Construction of the potential relationship between circRNAs and miRNAs. The green oval represents circRNAs. Possible paired miRNAs are represented by circles. The deeper the color, the more stable the binding between circRNAs and miRNAs.

For a deeper understanding of circRNAs’ capability in the restraint stress regulatory network in rats, the 10 circRNAs established in our research were predicted for circRNA-miRNA binding using miRanda software. A total of 285 circRNA-miRNA potential binding sites were identified. Using Cytoscape, we constructed a circRNA-miRNA interaction network for these 285 pairs (Figure 5). Notably, *circCACNA2D3(5,6,7,8)* demonstrates a high binding affinity with rno-miR-881-5p, rno-miR-871-5p, and rno-miR-743a-5p, and *circDCC(22,23,24,25)* exhibits a robust interaction with rno-miR-433-5p.

### 3.7 Abundance of validated circRNAs in different tissues

**Figure 6:**
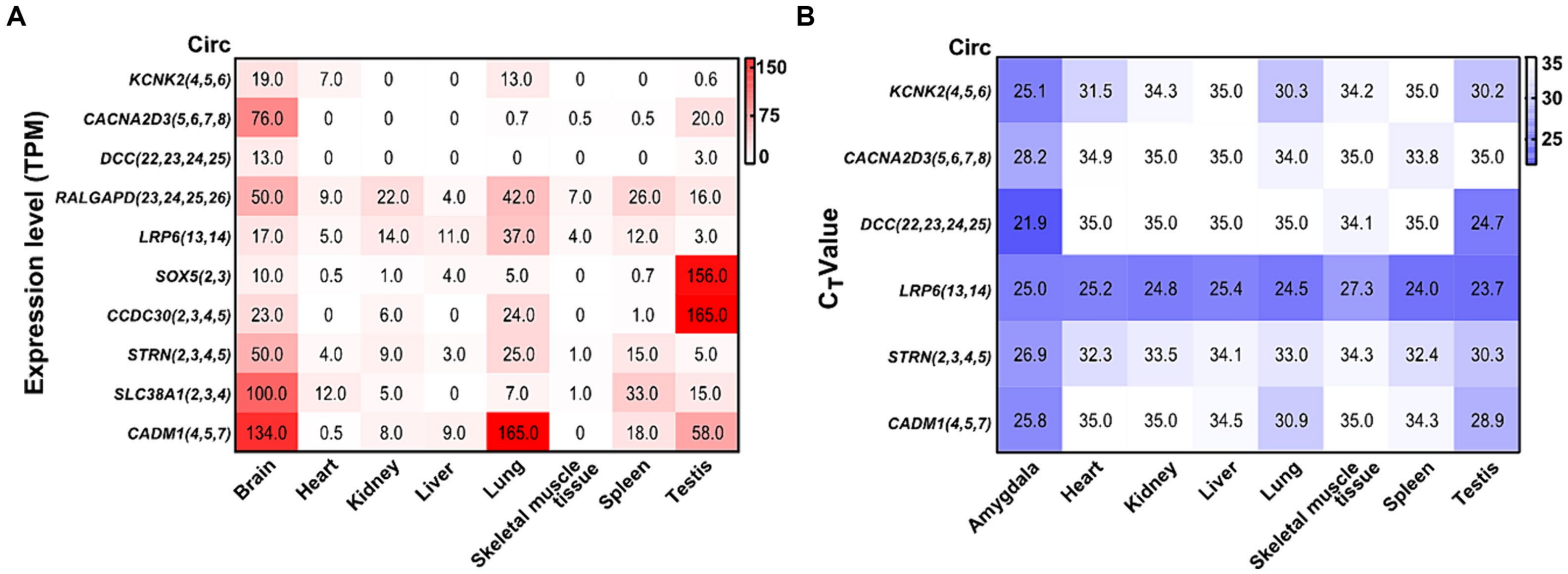
Heatmap of validated circRNAs in eight tissues and their expression characteristics. **(A)** Heatmap of the expression of parental genes of 10 circRNAs in the ensemble. White represents 0, indicating no expression in the tissue; red represents the expression level TPM; and the brightness of the color represents the degree of expression. **(B)** Heatmap of the expression of six validated circRNAs in eight tissues. White stands for undetermined, indicating no expression in tissue; purple represents the expression level (C_T_ value); and the brightness of the color represents the degree of expression.

By analyzing a total RNA sequencing dataset, the heatmap showed that circRNA expression had universal diversity in different tissues, whereas the brain showed a higher abundance in all tissues (Figure 6A). To screen circRNAs that could be specifically expressed in different tissues, we examined the expression of six circRNAs in eight tissues by qRT-PCR. CircRNA expression investigation revealed higher abundance and diversity in the amygdala, followed by the testis, lung, spleen, and heart. These results suggest that *circCACNA2D3(5,6,7,8)*, *circDCC(22,23,24,25)*, and *circCADM1(4,5,7)* are enriched in the brain compared with other tissues. Notably, *circLRP6(13,14)* was abundantly distributed in all eight tissues (Figure 6B).

## 4 Discussion

Short-term and moderate stress stimulation can enhance the body’s adaptability, whereas long-term and excessive stress can cause various disorders of physiological homeostasis and may even induce neuropsychiatric diseases, including depression, anxiety disorder, and PTSD (Ghosh et al., 2021; Zhang et al., 2021). The pathogenesis of restraint stress is complex and lacks an effective diagnosis and treatment. CircRNAs are extremely stable, genetically conserved, and prevalent in the mammalian brain (Rybak-Wolf et al., 2015; Sharma A. R. et al., 2021). Many studies have demonstrated that circRNAs are not only by-products of transcription, but also widely involved in the emergence and development of various neurological diseases (Dong et al., 2023; Zhou et al., 2024). To explore the profiles of circRNAs in restraint stress in the amygdala, we first validated that induced rats exhibited potent anxiety-like behaviors after restraint stress. Subsequently, we extracted total RNA from the rat amygdala, and RNA-seq was performed to compare the stress and control groups. After conducting bioinformatic analysis and validation trials, we observed that *circCACNA2D3(5,6,7,8)*, *circDCC(22,23,24,25)*, and *circCADM1(4,5,7)* were highly expressed in the amygdala under restraint stress. These findings provide novel and supplementary evidence for the probable interactions among circRNAs from the amygdala as potential biomarkers of restraint stress.

Our research revealed that restraint stress exposure significantly decreased spontaneous activity in the central area of the OFT, which is indicative of higher general anxiety (Sakhri et al., 2020; Strekalova et al., 2022). Furthermore, restraint stress-exposed rats showed reduced activity in the open arms of the EPM, implying an emotional state of anxiety (Song et al., 2021). Restraint stress decreases body weight gain and increases serum CORT levels in stressed rats. These trends were consistent with the experimental results for the same period (Xu et al., 2019). These findings further illustrate the generation of stress states and demonstrate that the restraint stress model was successfully established.

CircRNAs are widely expressed and evolutionarily conserved in multicellular organisms ranging from worms, fruit flies, and mice to monkeys and humans (Jeck et al., 2013; Wilusz, 2018; Kristensen et al., 2019). Previously, most studies on circRNAs focused on tumors. This study analyzed high-throughput sequencing and potential functional analysis of circRNAs in the amygdala, which is strongly associated with stress management and is pivotal in emotional control, fear, anxiety, and cognitive development (Janak and Tye, 2015; Ozawa et al., 2020). The RNA integrity number of every sequencing sample was greater than 8 (Walker et al., 2023). We obtained 53.38 G of clean data. The average proportion of Q30 was 91.45%. The ratio of mapped reads was between 93.00 and 93.77%. These results indicated that the data quality was consistent among the samples and that the RNA-seq data were credible. In total, 10,670 circRNAs were detected in rats’ amygdalas of the control and restraint stress groups. CircRNAs are normally derived from exonic, intergenic, and intronic regions of multiple species. Consistent with previous studies, most circRNAs in our research were derived from exonic regions (> 80% in each sample) (Xia et al., 2016). Among all the discovered circRNAs, the greatest distribution of circRNAs was observed on chromosome 1, whereas the least distribution of circRNAs was detected on chromosome Y, consistent with earlier reports that the number of circRNAs identified is proportional to the length of the chromosome. Principal component analysis revealed no overlap in the 95% confidence intervals between the two groups. These findings indicate that all samples within each group were consistent but distinct between the groups. Restraint stress for 2 days could induce a significant conversion in the overall expression level of circRNAs in the amygdala of rats.

We investigated circRNA expression profiles to analyze the changes in circRNA expression under restraint stress. Nine upregulated and five downregulated differentially expressed circRNAs were identified in the amygdala, nine of them were exon circRNAs, which was the most. Based on the sequencing results and estimated relevant function, we selected 10 circRNAs for further validation research. The size and base sequence of all selected circRNAs were confirmed to be consistent with the high-throughput sequencing results. Moreover, nine of 10 selected circRNAs showed the same expression trend as RNA-seq. The fold-change in circRNA expression between the different groups was less pronounced. The use of multiple reference genes can help us better understand the experiment. Six of the 10 circRNAs were significantly differentially expressed between the restraint stress and control groups. Considering that the neurological system is a relatively stable dominant system for regulating physiological activities, the overall change may not be drastic when the body experiences external damaging factors. Previous studies have verified that circRNAs are particularly enriched in the nervous system and accumulate with age. Another possibility is to analyze circRNA variation that may cause hysteresis compared to the pace of change within the behavior (Du et al., 2022; Dong et al., 2023; Hosaka et al., 2023). The long-term changes in circRNAs in the amygdala after restraint stress warrant further investigation.

With the evolving recognition of ceRNA networks in various diseases, research targeting ceRNAs rather than ncRNAs alone may become increasingly important (Gil-Jaramillo et al., 2023). Therefore, GO annotation and KEGG analysis were employed to investigate the functions of differentially expressed circRNAs. In the GO analyzes, the clustered terms of genes were neuron part, axon, and neuron projection, which were associated with restraint stress. The amygdala has a neurotransmitter imbalance and neuronal dysfunction during stress, and its molecular mechanism may be related to the differential expression of circRNAs (Gasparini et al., 2020). KEGG pathway analysis indicated that differentially expressed circRNAs were most significantly enriched in the cortisol synthesis and secretion. The enrichment of GABAergic synapse, glutamatergic synapse, cell adhesion molecules, and axon guidance pathways were particularly noteworthy.

CircRNA is a novel and significant component of ncRNA. They can regulate neurological function and act as miRNA sponges to regulate miRNA target gene expression by competitively combining with miRNAs in neurological diseases (Xie et al., 2017; Panda, 2018; Qi et al., 2021; Malviya and Bhuyan, 2023). Ultimately, our findings revealed 285 interactive relationships between miRNAs and circRNAs. One miRNA can interact with various circRNAs, and the same circRNA can be targeted by various miRNAs. Notably, *circCACNA2D3(5,6,7,8)* and *circDCC(22,23,24,25)* showed stronger combining capacity with some miRNAs.

Numerous circRNAs in rats have been reported to be common in mammals (humans and mice), and their expression in different tissues varies and is closely related to physiological functions (You et al., 2015; Zhou et al., 2018). The database showed that the TPM of all verified circRNA parental genes mentioned above was higher in the brain; some showed special expression in the testis tissue. The six confirmed circRNAs with significant differential expression were highly expressed in the amygdala and testis tissues, and certain circRNAs also showed specific expression in the amygdala, which was consistent with the above database. The *circDCC(22,23,24,25)* parental gene is deleted in colorectal cancer, which is widely expressed in the brain and normally regulates cell migration, synaptic functions, cell adhesion, and tissue morphology (Dun and Parkinson, 2017; Torres-Berrío et al., 2017; Glasgow et al., 2020). *CircCADM1(4,5,7)* is highly expressed in amygdala tissue, and its parental gene is cell adhesion molecule 1, which is involved in biological activities, including cytoskeleton construction, cell adhesion, and cell signaling (Yamada et al., 2013). *CircCACNA2D3(5,6,7,8)*, *circDCC(22,23,24,25)*, and *circCADM1(4,5,7)* are specifically expressed in the amygdala and may be crucial for its regulation. Further research on their role in restraint stress is necessary to clarify the mechanism of stress-induced amygdala damage.

This study has several limitations. First, we only identified differentially expressed circRNAs in the amygdala of rats after restraint stress; sequencing analysis of other brain regions may provide a more comprehensive explanation. In addition, the non-invasive sampling and real-time monitoring enhance the importance of investigating stress-related biomarkers in peripheral blood. Second, although we observed that some circRNAs may be correlated with restraint stress, the molecular mechanisms of circRNAs in restraint stress are poorly understood. Thus, the role of circRNAs in the rat amygdala requires further investigation.

## 5 Conclusion

In summary, the behavioral data demonstrate that 2 days of restraint stress results in anxiety-like behaviors in rats. The circRNA expression profile in the amygdala of control and restraint stress rats was characterized. Our sequencing analysis revealed differential expressions of circRNAs in the amygdala of rats subjected to restraint stress. The parent genes of these differentially expressed circRNAs are predominantly associated with neuronal activity and neurotransmitter transport. Moreover, *circCACNA2D3(5,6,7,8)*, *circDCC(22,23,24,25)*, and *circCADM1(4,5,7)* exhibited high specificity in the amygdala, which might be helpful for the diagnosis of restraint stress and therapy for neurological disorders and may be crucial for identifying new potential biomarkers. Further research on the functions of circRNAs is essential to better understand the mechanisms underlying the occurrence and progression of stress.

## Data availability statement

The datasets presented in this study can be found in online repositories. The names of the repository/repositories and accession number(s) can be found at: https://www.ncbi.nlm.nih.gov/, PRJNA1071307.

## Ethics statement

The animal study was approved by the Laboratory Animal Ethical and Welfare Committee of Hebei Medical University. The study was conducted in accordance with the local legislation and institutional requirements.

## Author contributions

CW: Data curation, Formal analysis, Investigation, Methodology, Writing – original draft, Writing – review & editing. QW: Data curation, Formal analysis, Investigation, Writing – original draft, Writing – review & editing. GX: Investigation, Writing – review & editing. ZS: Investigation, Writing – review & editing. DZ: Investigation, Writing – review & editing. CM: Project administration, Writing – review & editing. YL: Project administration, Writing – review & editing. DW: Conceptualization, Writing – review & editing. XZ: Conceptualization, Supervision, Writing – review & editing. BC: Conceptualization, Funding acquisition, Project administration, Writing – review & editing.
