# Supplementary material for: Circular RNA expression profiles and functional predication after restraint stress in the amygdala of rats
Source: Front Mol Neurosci. 2024 Apr 15;17:1381098. doi: 10.3389/fnmol.2024.1381098 (PMC11056511; doi:10.3389/fnmol.2024.1381098)
Supplement: Supplementary file 1 [file Image_1.PDF]

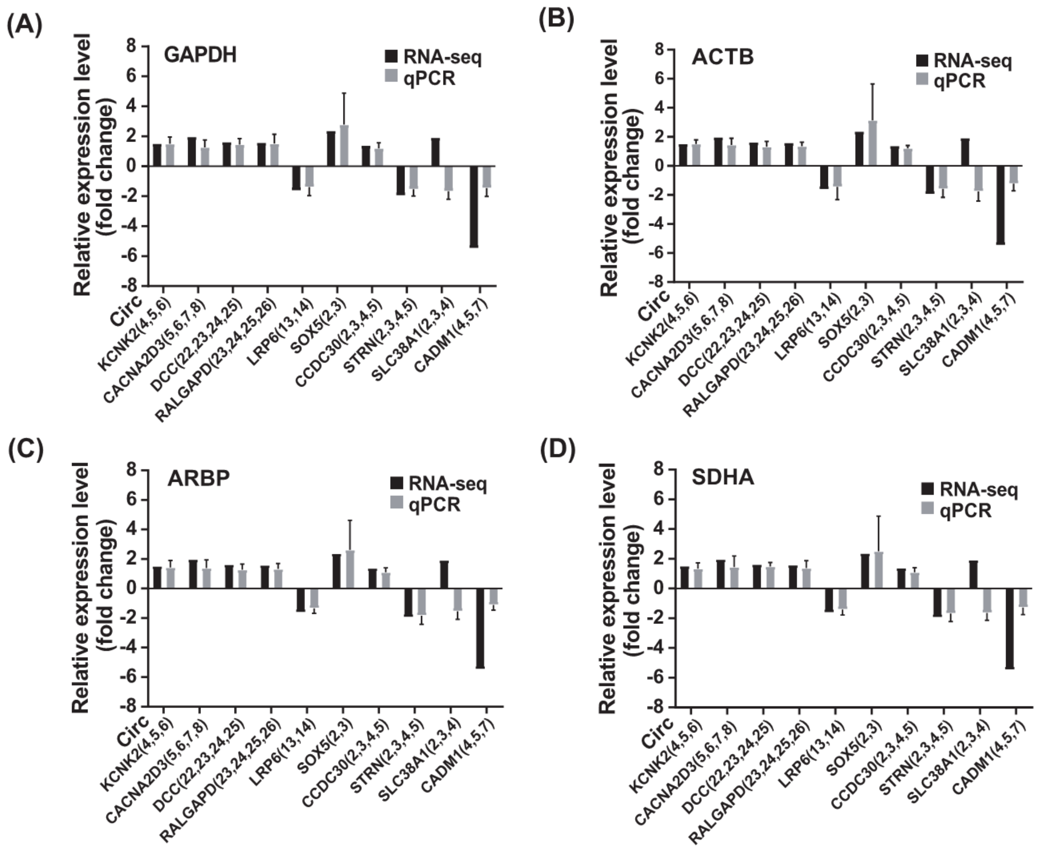


**Supplementary Figure S1** Comparison of the respective results from RNA-seq and qRT-PCR to verify the credibility of the RNA-seq analysis normalized by four control genes (GAPDH, ACTB, ARBP, and SDHA).
