# Supplementary material for: Circular RNA expression profiles and functional predication after restraint stress in the amygdala of rats
Source: Front Mol Neurosci. 2024 Apr 15;17:1381098. doi: 10.3389/fnmol.2024.1381098 (PMC11056511; doi:10.3389/fnmol.2024.1381098)
Supplement: Supplementary file 2 [file Image_2.PDF]

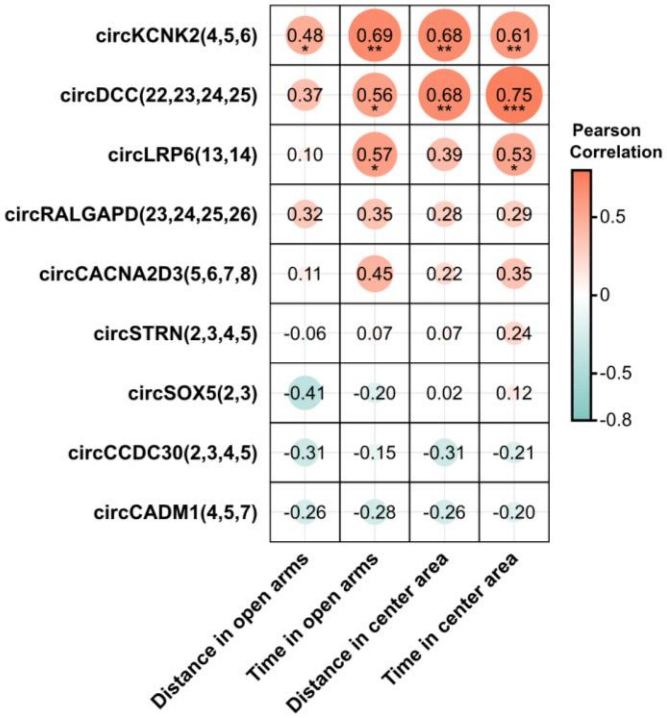

Supplementary Figure S2 Pearson correlation analysis between behavioral indicators and circRNAs expression levels in rat amygdala after restraint stress. *$P < 0.05$, **$P < 0.01$, ***$P < 0.001$.
